# Atomic force acoustic microscopy reveals the influence of substrate stiffness and topography on cell behavior

**DOI:** 10.3762/bjnano.10.223

**Published:** 2019-11-26

**Authors:** Yan Liu, Li Li, Xing Chen, Ying Wang, Meng-Nan Liu, Jin Yan, Liang Cao, Lu Wang, Zuo-Bin Wang

**Affiliations:** 1 Ministry of Education Key Laboratory for Cross-Scale Micro and Nano Manufacturing, Changchun University of Science and Technology, Changchun 130022, China; 2 International Research Centre for Nano Handling and Manufacturing of China, Changchun University of Science and Technology, Changchun 130022, China; 3 Computer Department, Changchun Medical College, Changchun 130031, China; 4 JR3CN & IRAC, University of Bedfordshire, Luton LU1 3JU, UK

**Keywords:** atomic force acoustic microscopy (AFAM), cell growth, nanopattern, stiffness, SU-8 photoresist, topography

## Abstract

The stiffness and the topography of the substrate at the cell–substrate interface are two key properties influencing cell behavior. In this paper, atomic force acoustic microscopy (AFAM) is used to investigate the influence of substrate stiffness and substrate topography on the responses of L929 fibroblasts. This combined nondestructive technique is able to characterize materials at high lateral resolution. To produce substrates of tunable stiffness and topography, we imprint nanostripe patterns on undeveloped and developed SU-8 photoresist films using electron-beam lithography (EBL). Elastic deformations of the substrate surfaces and the cells are revealed by AFAM. Our results show that AFAM is capable of imaging surface elastic deformations. By immunofluorescence experiments, we find that the L929 cells significantly elongate on the patterned stiffness substrate, whereas the elasticity of the pattern has only little effect on the spreading of the L929 cells. The influence of the topography pattern on the cell alignment and morphology is even more pronounced leading to an arrangement of the cells along the nanostripe pattern. Our method is useful for the quantitative characterization of cell–substrate interactions and provides guidance for the tissue regeneration therapy in biomedicine.

## Introduction

The interactions of cells with extracellular matrices (ECMs) play important roles in regenerative medicine and tissue engineering [1–2] as they affect many cell functions such as cell migration [3–4], attachment, proliferation [5–6] and differentiation [7–8]. Substrate stiffness and topography are two of the most important ECM physical parameters in regulating cell functions [9]. A previous study shows that cells attached to rigid substrates can spread thereby increasing the interaction area with other cells, while cells remain quiescent and are observed to be more spherical when attached to softer substrates [10]. Additionally, cells also show a tendency to migrate in the direction of increasing stiffness [3]. So far, the areas in which such behavioral patterns are induced are of several tens of micrometers, and it is still a challenge to produce patterns on the nanometer scale [11–13]. Controlling the substrate topography has effects on cell properties such as the cell alignment, cell shape and the distribution of molecules. However, many native tissues are not homogeneously stiff and it is not clear whether the controlled presentation of rigid and flexible material axes on the substrate governs the cytoskeletal and nuclear morphology [14].

Several techniques such as fluorescence microscopy [14–15], confocal microscopy, scanning electron microscopy (SEM) [12] and atomic force microscopy (AFM) [16–17] have been employed to investigate cell–substrate interactions. Fluorescence and confocal microscopy are traditional techniques to investigate the intra- and intercellular processes in biological studies, but the spatial resolution is poor [18]. SEM is capable of detecting the surface features of substrates and cells on the nanoscale, but the sample preparation is time-consuming and complex [19]. AFM is emerging as a valuable tool for true atomic resolution imaging [20] and is widely used in biomechanical studies [21]. Atomic force acoustic microscopy (AFAM) is a technique based on AFM for nondestructive imaging. This technique operates on a dynamic mode in which the AFM cantilever vibrates upon ultrasound excitation. Accordingly, AFAM shows the ability to measure nanomechanical properties and is an effective tool to measure soft materials such as biomedical tissues and polymers [18]. A number of research groups have reported that the AFAM method can characterize mechanical properties of buried structures. For example, the influence of the thickness on the elastic properties of porous nanofilms was evaluated by AFAM [22]. Periodical stiffness variations caused by molecular chains of copolymers could be observed by AFAM even when covered by soft layers [23].

**Figure 1:**
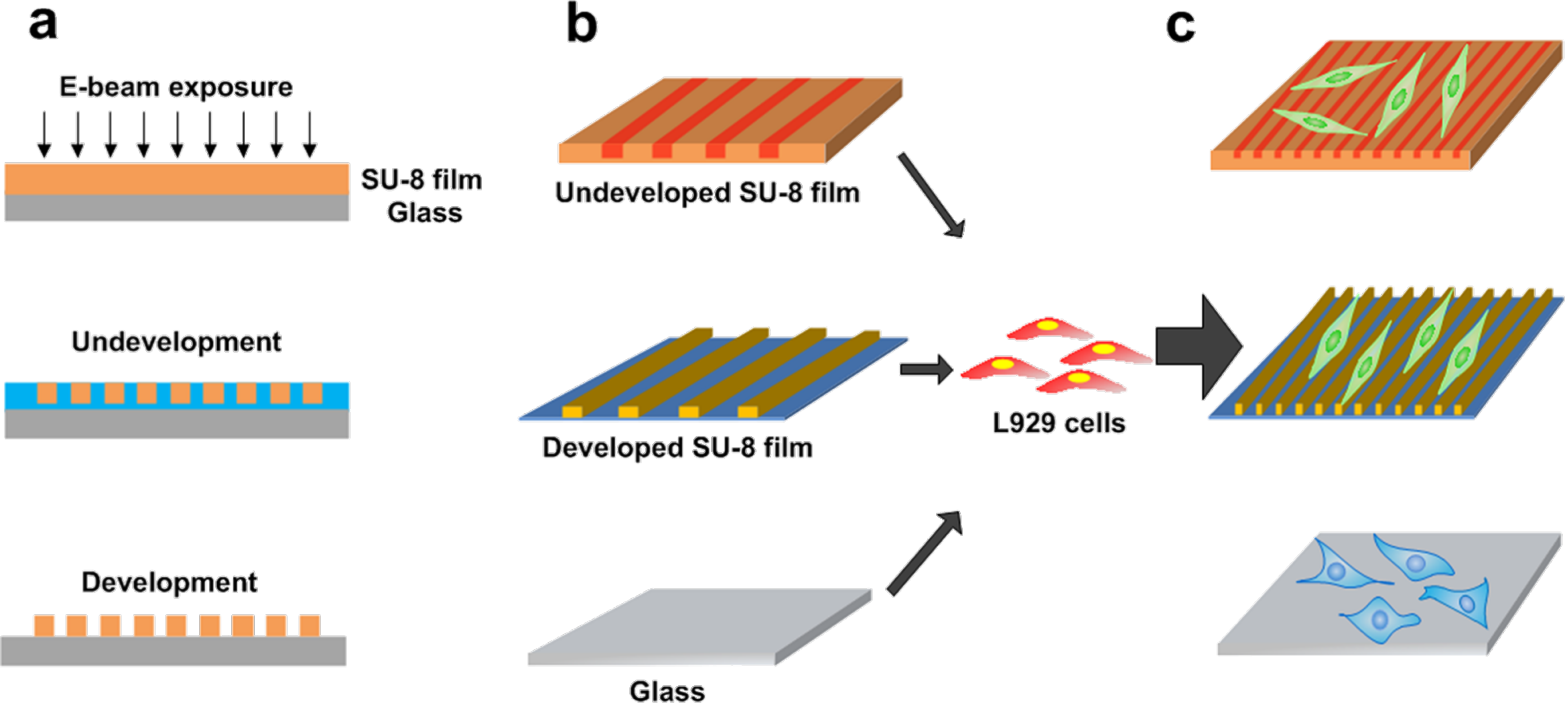
Fabrication of SU-8 films and differentiation of L929 cells cultured on the surfaces. (a) The fabrication process for producing tunable stiffness and topography substrates. (b) The undeveloped and developed SU-8 films and the reference glass substrate. (c) The changes in cellular behavior in response to the different substrates.

In this work, using AFAM, we aimed to study cell responses on stiffness and topography changes patterned onto a substrate. We used SU-8 photoresist films as the substrate and generated local changes in the stiffness and the nanopattern topography on the surface. The SU-8 photoresist has been used as the material for biosensors in living tissues [24] and cell culture molds in vitro due to its excellent biocompatibility [16] and mechanical strength [25–26]. The patterned stiffness of the SU-8 films was induced by electron beam lithography (EBL). The approach to control the stiffness and the topography of the substrate is shown in Figure 1. The rigidity of the film was tuned by varying the electron beam dosage, while the surface topography is determined by both the exposure dose and the development of the SU-8 films. We cultured L929 cells on undeveloped and developed SU-8 surfaces as well as on a reference glass substrate. The structural responses of the L929 cells on the substrate topography were probed using AFAM. A fluorescence microscope was also used to analyze the resulting cell morphology and alignment.

## Experimental

### Fabrication of SU-8 films as the stiffness and topography layers

The SU-8 films on glass slides were prepared as follows. At first, glass slides (10 mm × 10 mm) were cleaned by sonication in ethanol, then washed with deionized water and dried in air. The SU-8 photoresist (2000.5, MicroChem) was spin-coated on the glass slides at a speed of 4000 rpm for 60 s. The slides were subsequently heated to 95 °C on a hot plate for 60 s to remove the solvent. The resulting SU-8 coating is approximately 400 nm thick (Supporting Information File 1, Figure S1), as measured by SEM (FEI Quanta 250 FEG, USA).

The SU-8 films were then patterned by EBL using a nanopattern generation system (NPGS, V9.1, from JC Nabity Lithography Systems). The SU-8 films were exposed to the 30 kV electron beam at a beam current of 63 pA and a spot size of 2.5 nm. In order to fabricate the stripe pattern over a large area (1 mm × 1 mm), 2 × 2 arrays of 500 μm each were patterned. To produce so-called patterned stiffness surfaces, we employed exposure doses of 200, 500, 1000, 2000 and 5000 μC∙cm^−2^ to SU-8 films and left them undeveloped. By culturing L929 cells on such exposed but undeveloped SU-8 films, we found that exposure doses lower than 200 μC∙cm^−2^ should not be used because the cells cannot survive on the corresponding surfaces. To obtain so-called topography surfaces, the SU-8 films were first exposed to EBL at the optimum exposure dose of 1000 μC∙cm^−2^, which produced the best topography structure. Then the SU-8 films were developed for 1 min using the MicroChem SU-8 developer and dried in air. The resulting developed nanostripe sections are separated by 1.5 μm and have of a width of 500 nm and a deepness of 175 nm.

### AFAM imaging

Atomic force acoustic microscopy (CSPM5500, Benyuan, China) was used for the imaging the SU-8 surfaces and the L929 cells cultured on the different substrates. The specimen to be studied was mounted on the top of an ultrasonic transducer by a coupling medium (Vaseline). The ultrasonic transducer under the sample holder was used to generate ultrasound waves propagating into the sample. An AFM tapping probe (Tap300Al-G, BudgetSensors) was used in all imaging experiments. A lock-in amplifier (SR830DSP, Stanford Research Systems, USA) was employed to analyze the cantilever oscillation signals. When the probe tip scanned across the specimen surface, acoustic and morphological images were acquired simultaneously. The deflections of the cantilever represent the morphological information, and the vibrations of the cantilever correspond to the acoustic signals. By employing a suitable mechanical model to describe the vibrations of the cantilever, the contact stiffness of the tip–sample interaction can be estimated [27–28]. For one-phase homogeneous materials, the detected vibrations will remain relatively uniform, while for inhomogeneous materials, the vibrational amplitude depends on the elastic properties and the AFAM image will reflect the stiffness changes [29].

After parameter optimization, we selected a resonance frequency of 38.41 kHz and a reference point value of 0.1 V. To ensure comparability of the stiffness measurements of the different SU-8 substrates, the acoustic images were recorded using the same parameters.

The Young’s moduli of the fabricated substrates were evaluated by fitting the force–distance curves with a Hertzian cone. A probe sensor (ContAl-G, Budget Sensors) was used in the force modulation mode for measuring the Young’s moduli. The cantilever spring constant was 0.2 N∙m^-1^. For each film, about 20 force curves were obtained around the stripe and the unexposed region of the patterns.

The root-mean-square roughness of the SU-8 films was determined from the surface morphological images of AFAM. The scan was performed on a field of view of 10 μm × 10 μm at the frequency of 1 Hz for surface roughness measurements.

### Cell culture

The L929 cells from the mouse fibroblast cell line were cultured at 37 °C in a minimal essential medium (MEM, Solarbio) supplemented with 10% fetal bovine serum. Before seeding, the SU-8 substrates and the reference glass substrate were placed into a 100 mm cell culture well. After sterilization for 1 h with ultraviolet light, the substrates were rinsed thrice with phosphate-buffered saline (PBS) and once with the cell-culture medium. Then, the cells (approximately 1 × 10^5^ cells·mL^−1^) were seeded on the fabricated SU-8 substrates and the reference glass substrate.

After 48 h of cell culture, prior to the AFAM measurements, the L929 cells were washed three times with PBS. Then, the cells were treated with 4% (w/v) paraformaldehyde solution for 15 min and washed with deionized water. After being dried in air, the L929 cells were measured by AFAM at room temperature.

### Immunofluorescence and image analysis

After 48 h, the L929 cells were fixed with 4% (w/v) paraformaldehyde solution for 15 min. Subsequently, the cells were washed three times with PBS. The cytoskeletons of the L929 cells were stained green by phalloidin (Alexa Fluor 488) for 15 min, and the nuclei were stained blue by DAPI for 5 min. Finally, after washing once with PBS, the L929 cells were observed with a fluorescence microscope (Nikon ECLIPSE Ti). The images were captured using a Nikon DS-Ri2 camera, and imaging was repeated at least four times for each group. The NIH Image J software [30] was used to analyze the images and quantify the orientation angle and the cell areas. For each experiment an average of 150–200 cells were counted.

## Results and Discussion

### Characterization of substrate morphological and mechanical properties by AFAM

Here, we used AFAM to obtain quantitative information on undeveloped and developed SU-8 film surfaces. AFAM uses the near-field detection of acoustic signals to study surfaces and even buried structures [31–32]. For AFAM, a transducer generates ultrasound waves propagating into the sample to cause vibrations of the sample surfaces [29,33]. When the probe sensor is in contact with the sample surface, the AFM cantilever directly reflects the vibrations. By modulating the drive frequency and the excitation amplitude used for AFAM imaging, the cantilever is set to adopt to the feedback signal. Finally, by analyzing the measured vibrational amplitudes and morphology signals, the acoustic and morphological images can be reconstructed.

**Figure 2:**
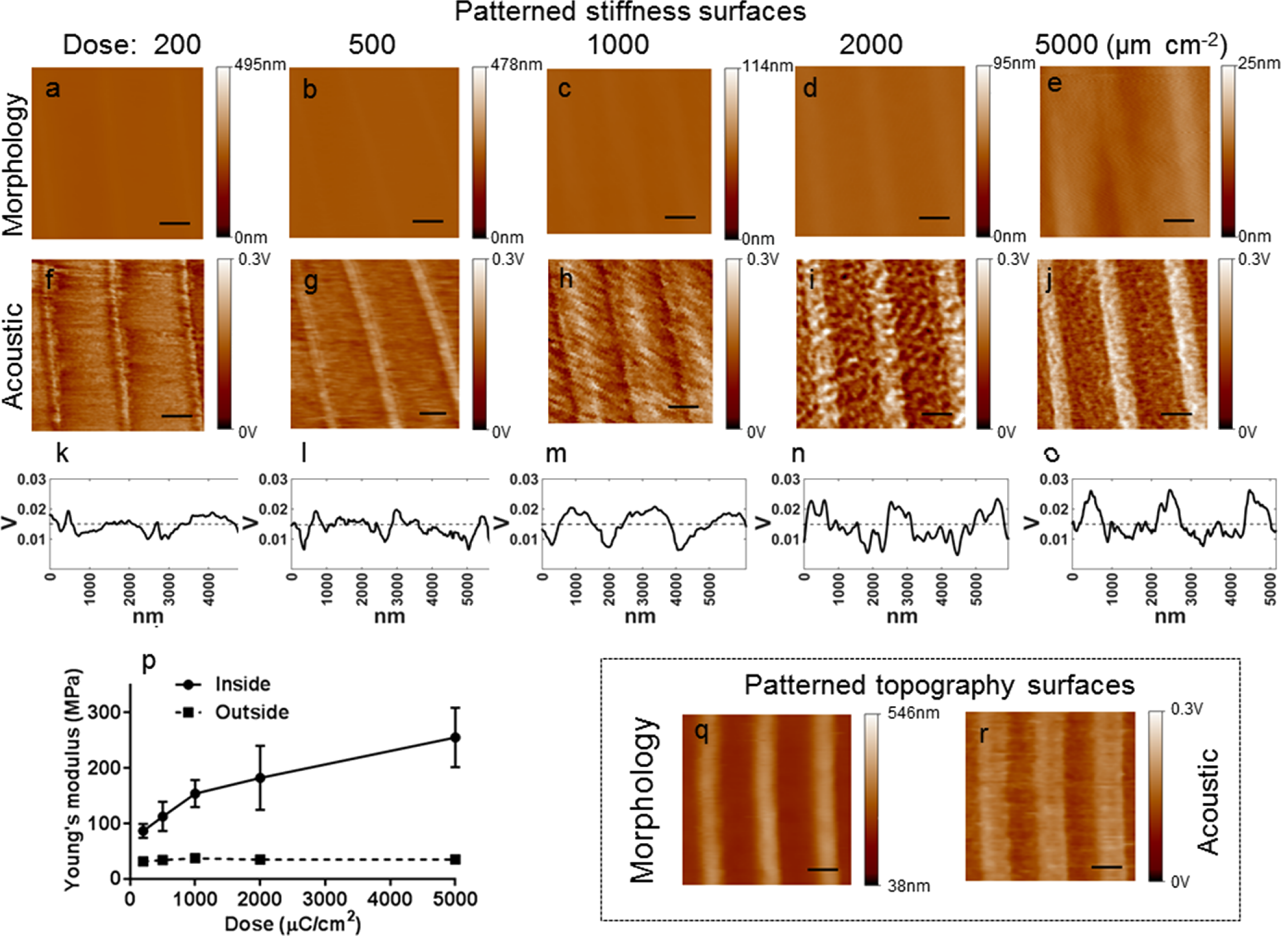
AFAM characterization of the SU-8 film surfaces. Morphological images (a–e) and acoustic images (h–j) of the exposed and undeveloped SU-8 film surfaces (patterned stiffness surfaces) exposed to EBL at exposure doses of 200, 500, 1000, 2000 and 5000 μC cm^−2^, respectively. (k–o) Vibrational amplitudes in cross sections of the acoustic images (p) Young's modulus values of the undeveloped SU-8 arrays at different exposure doses.; “inside” designates the bright regions (solid line) and “outside” corresponds to the dark regions (dash line). Morphological image (q) and acoustic image (r) of the patterned topography surface at an exposure dose of 1000 μC cm^−2^. Scale bars: 1 μm.

Figure 2a–j shows the AFAM images of the patterned stiffness surfaces (SU-8 films exposed to EBL and left undeveloped) after exposition to EBL at different exposure doses. Apparently, stripe patterns are observed in all acoustic images while in the morphological images the films appear nearly flat. The line widths of the nanopatterns observed in the acoustic images are slightly different, but the periodicities are almost the same (≈2 μm). The bright and the dark regions in the acoustic images correspond to the stripes and the unexposed regions on the patterned surfaces, respectively. The dark areas in the acoustic images have small vibrational amplitudes compared to the bright areas. According to the images obtained, the measured amplitudes of the areas related to the stripes on the developed films are clearly higher than those of the unexposed regions. Figure 2k–o shows the responses of the cantilever to changes of the vibrational amplitude corresponding to cross sections of the acoustic images. Using the same resonance frequency (38.41 kHz), we observe an increase of the image contrast with increasing exposure dose. This could be attributed to the increase of the surface stiffness. To further characterize the elasticity of the substrate the Young’s moduli of the undeveloped arrays were calculated using the Hertzian model. Here, the Young’s modulus measurements were repeated twenty times on each sample around the stripes and the unexposed regions of the patterns. A similar trend for surface stiffness is observed as plotted in Figure 2p. The plotted curves indicate a significant change of the Young´s moduli of the stripes on the undeveloped arrays from 870 ± 12 to 255 ± 53 MPa, while the Young´s moduli of the unexposed regions change by only about 35 ± 1 MPa. Moreover, the Young’s moduli of the stripes are much higher than those in the unexposed regions. These results show that the EBL exposure dose largely determines the stiffness of the stripes, but not that of the unexposed regions. In summary, in the acoustic images we observe areas with higher vibrational amplitudes corresponding to stiffer regions on the SU-8 films. Indeed, the different responses observed in the morphological and the acoustic images can be attributed to stiffness changes of the patterned stiffness surfaces. Even at unsuitable exposure doses at 200 and 500 μC·cm^−2^ which polymerization of the SU-8 resist cannot be completed, the pattern of stiffness is still enhanced.

In the next step, after the SU-8 film had developed for 1 min, we chose the optimum exposure dose of 1000 μC∙cm^−2^ to obtain strip-patterned topography surfaces. The AFAM images of these topography surfaces are shown in Figure 2q and Figure 2r. The morphological and the acoustic images are clear. Yet, the acoustic image reveals large edges resulting from the different stiffness of the stripes and the unexposed regions. Finally, the acoustic images depict more details of the real nanostructures at higher contrast and lower noise.

### Influences of substrate stiffness on L929 cell morphology and migration

**Figure 3:**
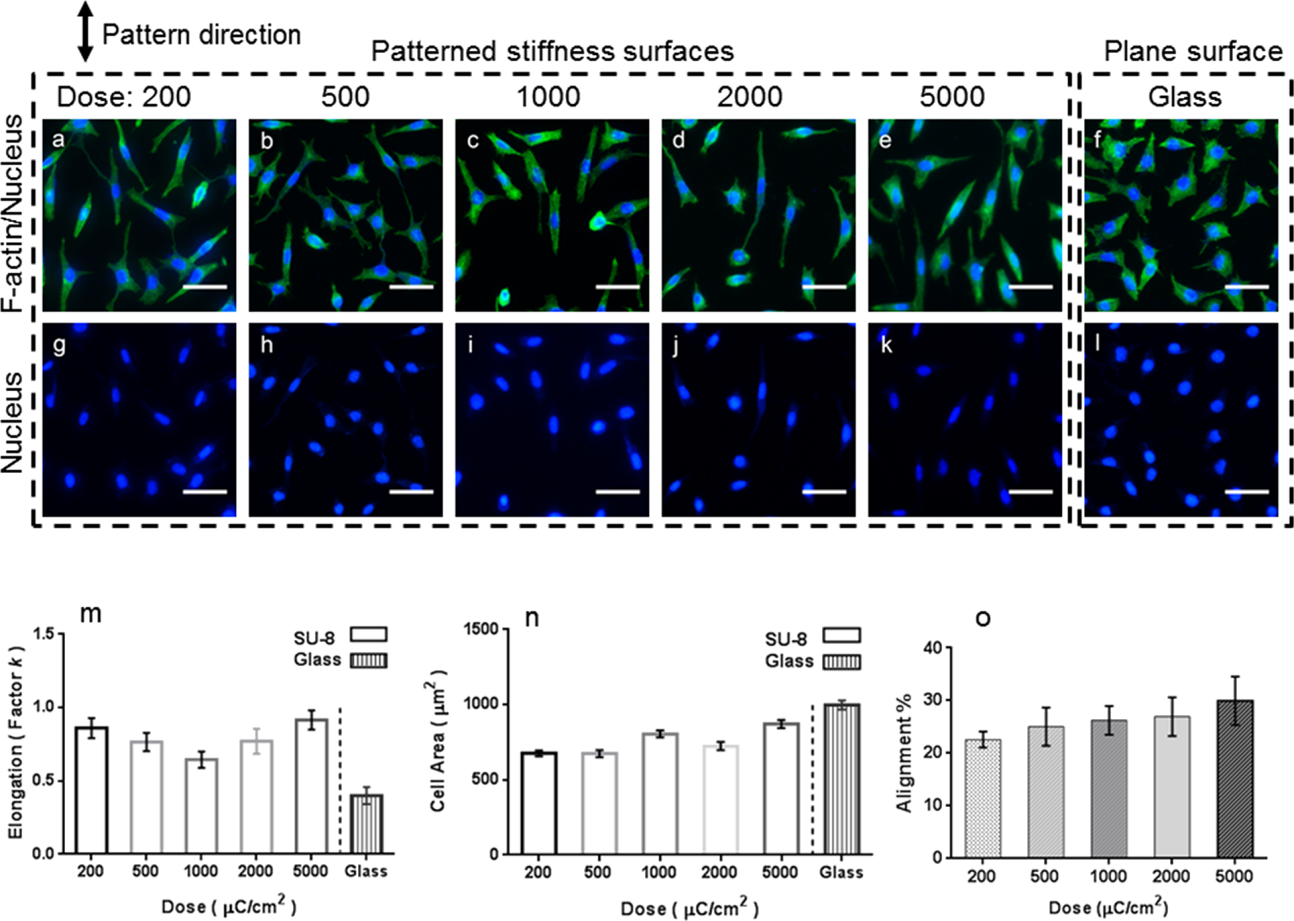
Comparison of the growth of the L929 cells on the SU-8 substrates of varying stiffness and on the reference glass substrate. Fluorescence micrographs of the L929 cells with stained F-actin (a–f) and stained cell nuclei (g–l) on the undeveloped SU-8 films exposed to different EBL doses and on the reference glass substrate. The stripes are vertically aligned. The cell nuclei were stained blue with DAPI, and F-actin was stained green with Alexa Fluor 488. (m) Elongation of the cells on the patterned stiffness surfaces and on the reference glass substrate. (n) Size of the cells on the patterned stiffness surfaces and on the reference glass substrate. (o) Alignment rate (alignment %) of the cells on the patterned stiffness substrates. (Data are expressed as the mean ± standard error of mean (s.e.m.) of four samples, *n* = 50 cells). Scale bars: 20 μm.

After substrate fabrication, we cultured L929 cells on the undeveloped SU-8 films of different stiffness and on a reference glass substrate. Fluorescence microscopy was used to investigate the role of the substrate stiffness in controlling the cell properties, including the size of the cell areas and the alignment of adherent cells (Figure 3a–l). We examined the morphology of the L929 cells by quantifying the cell spreading areas and the cell elongation. The captured images demonstrate that after 48 h of cell seeding, the L929 cells on the patterned stiffness films are significantly elongated compared to those on the reference glass substrate. The nuclei of the cells on the patterned stiffness surfaces are also deformed (Figure 3g–l). It has previously been reported that mechanical tension causes the changes of cell shape and the nuclear deformation [34]. Thus, we conclude that the surface stiffness can affect the morphology of the L929 cells, which further influences the distortion of the nuclei.

To further quantify the elongation of the cells we calculated the ratio of the nuclear axes expressed by the elongation factor *k*. This factor is equal to the length of the long axis divided by that of the short axis minus one [30]. For example, *k* of a circle is 0, and it is larger than 0.4 for an elongated structure. From Figure 3m and Figure 3n, we infer that both the elongation and the area spread of the L929 cells on the undeveloped SU-8 films remain statistically unchanged in the stiffness range investigated. These results suggest that modulating the EBL exposure doses does not significantly influence the adhesion and the spreading of the L929 cells on the patterned stiffness surfaces. This agrees well with the recent finding that changes in the cell shape cannot be attributed to changes of the substrate matrix elasticity [35]. However, the spreading and the differentiation of hepatic stellate cells (HSCs) have been related to the local stiffness of a substrate with a stiffness pattern size of 200 μm. On the other hand, no spreading or differentiation of HSCs were observed on substrates with patterns smaller than 200 μm (for instance, 50 and 100 μm) [10].

Moreover, we quantified the alignment rate (alignment % in Figure 3o) of the cells as a function of the different exposure doses. The determination of the nuclear alignment angles and the cell counting for angles smaller than 15° between the long axis and the grating were performed by using the Image J software. Figure 3o shows that the cells grown on the undeveloped surfaces are slightly more aligned to the stiffer axis ((22.6 ± 1.521)% to (29.97 ± 4.57)%). In contrast, the L929 cells grown on the reference glass substrate appear disorganized and have no preferential orientation (Figure 3f–l). These results suggest that the stiffness of the stripe patterns significantly affects the cell alignment, and a pronounced contrast of high and low stiffness regions on the surface is ideal for cell alignment [5]. Hence, the patterned stiffness does not to significantly impact the cell morphology, but it influences the cell rearrangement. However, because chemical surface treatments have been reported to support the cell spreading [36], the small changes observed here may be due to the littleness of the surface modifications. A recent study documents that protein coating or oxygen plasma treatment of substrate surfaces may influence the phenotypic equilibrium of cells [37].

### L929 cell responses to the topography substrate

**Figure 4:**
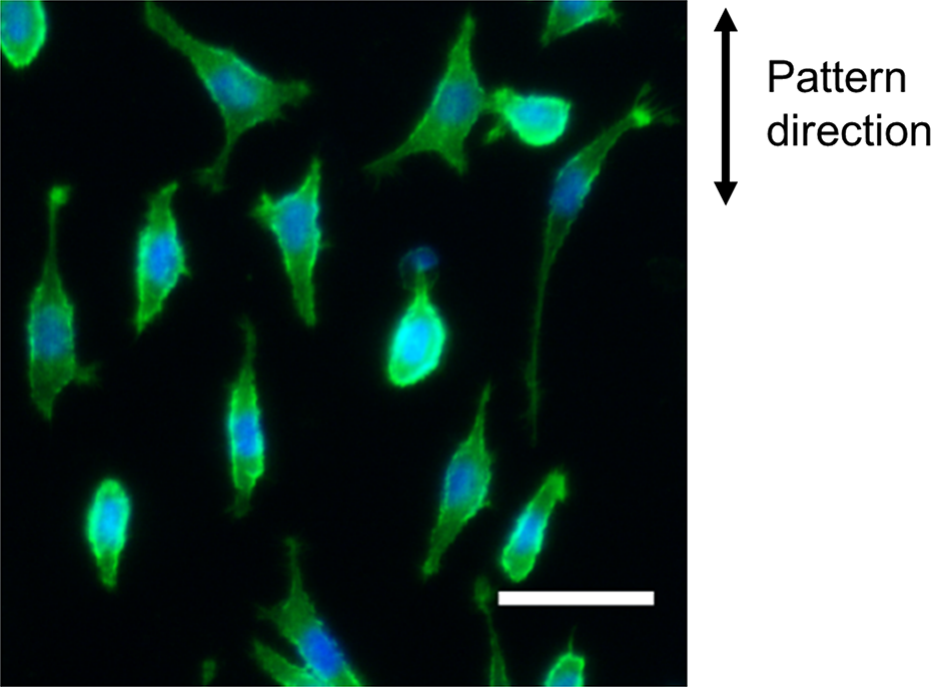
Fluorescence micrograph of the L929 cells cultured on the topography substrate (developed SU-8 surface). Scale bar: 20 μm.

We also seeded L929 cells on the topography surface, the developed SU-8 surface exposed to EBL at an exposure dose of 1000 μC∙cm^−2^. Figure 4 shows the fluorescence micrograph of the L929 cells on the topography surface. Our study was conducted using stripes patterns of sub-micrometer size. Figure 4 shows that the cells on the developed substrate cannot spread freely and become elongated in the stripe direction. The alignment and elongation of the L929 cells are attributed to the topographical pattern. Similar responses to the patterned topography have been reported for other types of cells. Biela et al. reported that human fibroblast cells were most sensitive to groove patterns and acted as triggers for the alignment of endothelial and smooth muscle cells [38]. Furthermore, osteoblast cells were sensitive to a line pattern but not to any other patterns (dots, holes or hexagons) [39].

### Influences of stiffness and topography on L929 cell responses by AFAM imaging

**Figure 5:**
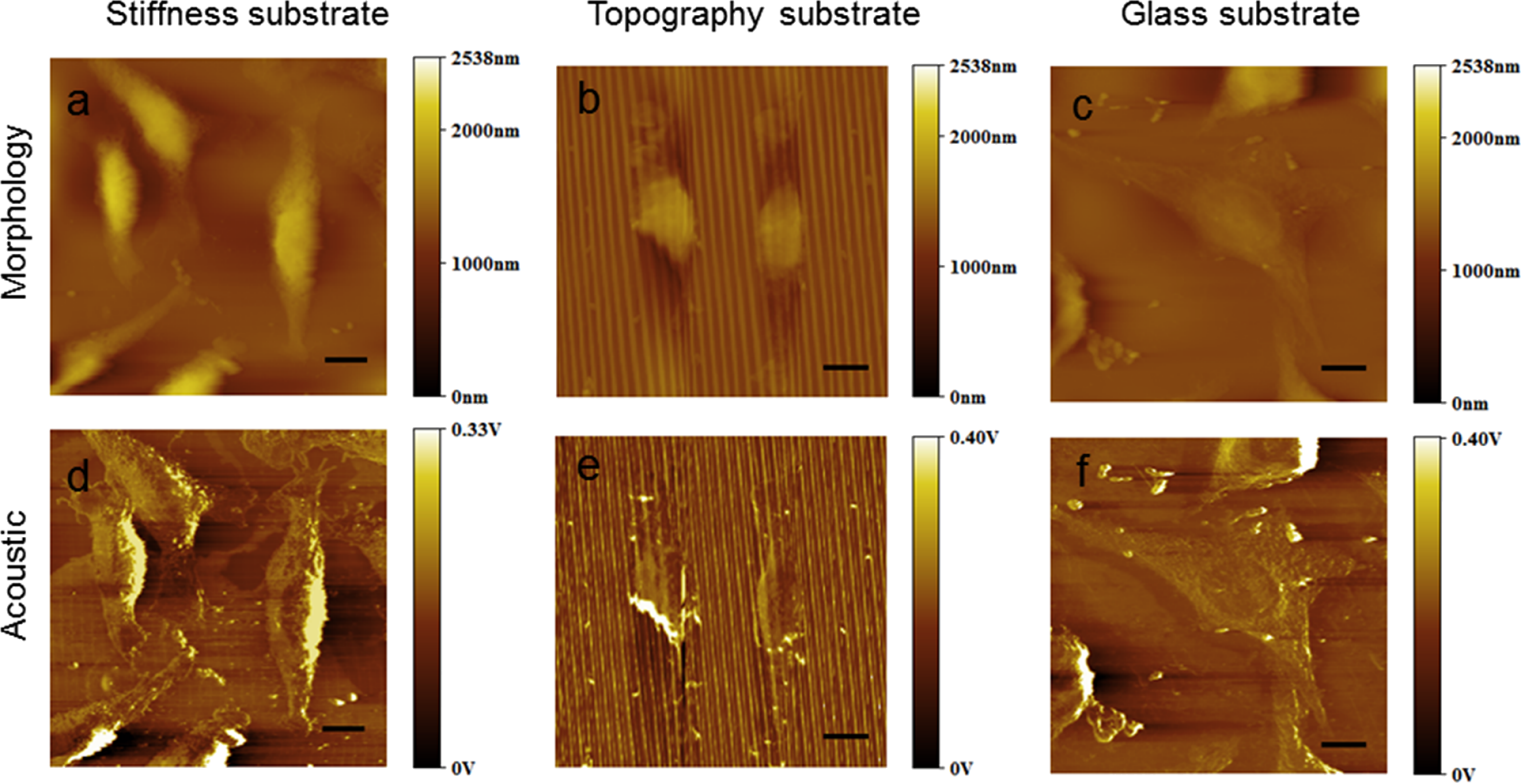
AFAM images of the L929 cells cultured for 48 h on the patterned stiffness substrate (a, d), on the patterned topography substrate (b, e) and on the reference glass substrate (c, f). Scale bars: 10 μm.

To further assess the fidelity of cell attachment to each substrate on the nanoscale, the L929 cells cultured on different substrates were imaged using AFAM. Figure 5 shows that the cells present a spindle shape on both the patterned stiffness substrate and the topography substrate. From the acoustic images, we can clearly see that the L929 cells are arranged along the direction of the stripe patterns on the stiffness and topography substrates. In addition, the cellular pseudopods are oriented in the same direction. In contrast, the cells on the reference glass substrate mainly have a flat stellate shape characterized by a big nucleus and increased cell spreading. These results disagree with the recent finding that the cell spreading on a SU-8 substrate was larger than on glass [16]. However, the SU-8 substrate studied in [16] had no patterns imprinted. Further studies demonstrate that cells trap into along the grooves or the edges of a micropattern [40], while on a SU-8 surface with a nanopattern cells are restricted by the pattern and spread across the grooves [41]. Thus, we believe that the nanopattern, although too small to restrict the cell growth, may enhance the elongation of the cells and affect the cellular arrangement. Moreover, the surface roughness of various samples was investigated using the morphological AFAM images (Supporting Information File 1, Figure S2). The roughness was determined as 0.6–5.6 nm for the undeveloped surfaces and 101–141 nm for the developed surface. Roughness values around 100 nm have been reported to significantly restrict the spreading and the orientation of cells [42]. This is in agreement with a recent study concluding that the surface roughness of substrates would affect the adhesion and the morphology of cells [43].

**Figure 6:**
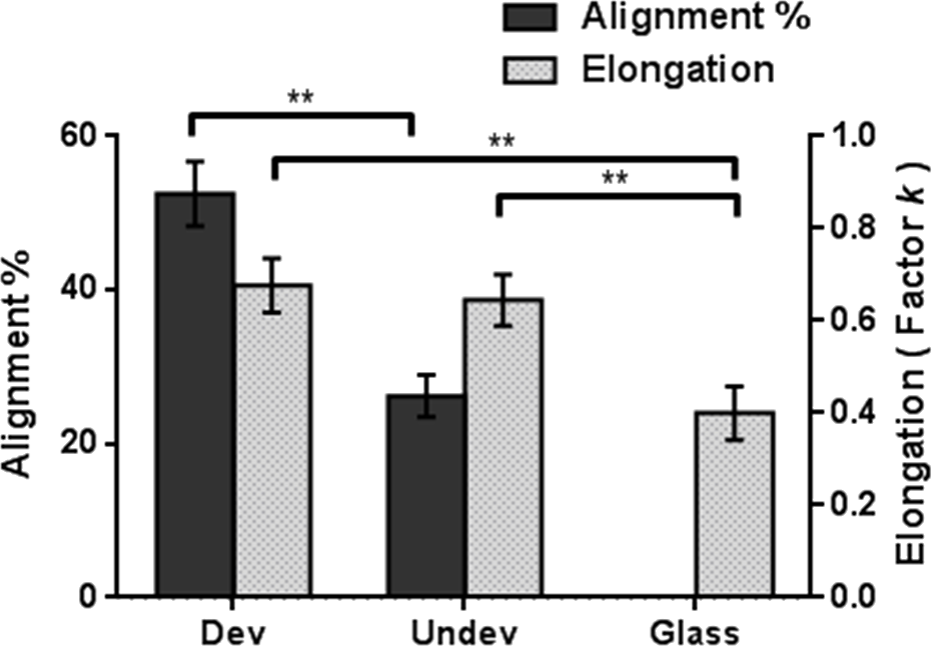
The alignment rate (alignment %) and the elongation factor of the L929 cells cultured on the developed and undeveloped SU-8 films and on the reference glass substrate. Data are expressed as mean ± s.e.m. of four samples, *n* = 150 cells, ** *p* < 0.01.

To assess how the stiffness and the topography differ in influencing the cells, the alignment rates and the elongation factors of the L929 cells cultured on the three substrates have been determined as shown in Figure 6. The cytoskeletal alignment rate of the cells on the undeveloped SU-8 substrate (26.247 ± 2.745%) is significant but much less pronounced than that of the cells on the developed SU-8 substrate (52.535 ± 4.198%). The elongation factor *k* determined for the cells on the developed SU-8 substrate (0.677 ± 0.058) is slightly higher than that on the developed SU-8 substrate (0.645 ± 0.055), while it is only 0.4 ± 0.058 on the reference glass substrate. Finally, we find that the substrate stiffness only somewhat modulates the morphology of L929 cells, while the substrate topography drastically impacts on the cell morphology yielding cells of organized cytoskeletons and elongated shapes. We confirm that substrate topography is a crucial parameter to regulate the L929 cell alignment and morphology, and substrate stiffness is less decisive. In line with previous studies [38,44–45], the groove topography can have dramatic effects on the alignment and the elongation of various types of cells. The groove topography can precisely control the morphology and the orientation of fibroblast cells, and the effects of nanotopographic substrates are greater than those of microtopographic ones [44]. Yet, the stiffness of the substrate also regulates the cell morphology and movement. It has been observed that the fibroblasts cultured on the micropatterned film move towards stiffer surfaces in the stripe pattern, particularly at the boundaries between a stiff area and a soft area [46]. However, there are only few studies on the spreading of fibrotic cells on nanopatterned stiffness substrates. In this work, we also investigated the impact of substrate stiffness and topography on the spreading of L929 cells. In summary, our study broadens the understanding of the influence of a nanopatterned substrate on the behavior of L929 cells.

## Conclusion

The stiffness and the topography of ECMs are important for cell behavior, but there is still a lack of effective methods to characterize these features. Therefore, we examined the properties of patterned substrates and the behavior of cells cultured on the substrates using AFAM. Using EBL we imprinted nanoscale patterns of tunable stiffness and topography on SU-8 films and investigated the responses of the morphology of the L929 cells cultured on them. The acoustic images of the undeveloped patterned SU-8 films (patterned stiffness surfaces) clearly indicate an increase of their surface elastic deformation with increasing EBL exposure dose. Fluorescence microscopy images show that the L929 cells cultured on the patterned stiffness surfaces have a modified shape. However, the cell shape does not change as a function of the different elasticities. Furthermore, the L929 cells align along the stripes of the patterned stiffness surfaces, and a higher mechanical contrast between the stripes and the background of the substrate surfaces is found ideal for the cell alignment. The spreading of the cells cultured on the developed patterned SU-8 film (topography substrate) is strongly restricted by the pattern, and the cells are elongated along the stripe direction. Interestingly, at the nanoscale, the L929 cells appear to respond more strongly to the topography patterns than to the stiffness patterns. Finally, our findings illustrate a method to transfer the nanostructural and mechanical properties of the substrate to the cells. This approach is useful for the investigation of biological processes, tissue development and cell-based regenerative medicine.

## Supporting Information

The Supporting Information features measurements of the SU-8 coating thickness and the surface roughness.

File 1 Additional experimental data.
