# Supplementary material for: Atomic force acoustic microscopy reveals the influence of substrate stiffness and topography on cell behavior
Source: Beilstein J Nanotechnol. 2019 Nov 26;10:2329–37. doi: 10.3762/bjnano.10.223 (PMC6902897; doi:10.3762/bjnano.10.223)
Supplement: File 1 — Additional experimental data. [file Beilstein_J_Nanotechnol-10-2329-s001.pdf]

BEILSTEIN JOURNAL OF NANOTECHNOLOGY

Supporting Information

for

Atomic force acoustic microscopy reveals the influence of substrate stiffness and topography on cell behavior

Yan Liu, Li Li, Xing Chen, Ying Wang, Meng-Nan Liu, Jin Yan, Liang Cao, Lu Wang and Zuo-Bin Wang

Beilstein J. Nanotechnol. **2019,** *10,* 2329–2337. doi:10.3762/bjnano.10.223

Additional experimental data

License and Terms: This is a supporting information file under the terms of the Creative Commons Attribution License (http://creativecommons.org/licenses/by/4.0). Please note that the reuse, redistribution and reproduction in particular requires that the authors and source are credited.
The license is subject to the *Beilstein Journal of Nanotechnology* terms and conditions: (https://www.beilstein-journals.org/bjnano)

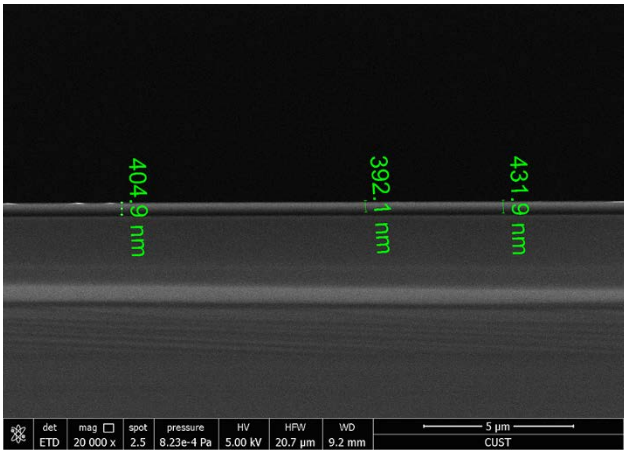

Figure S1: Coating thickness measurements of SU-8 by SEM.

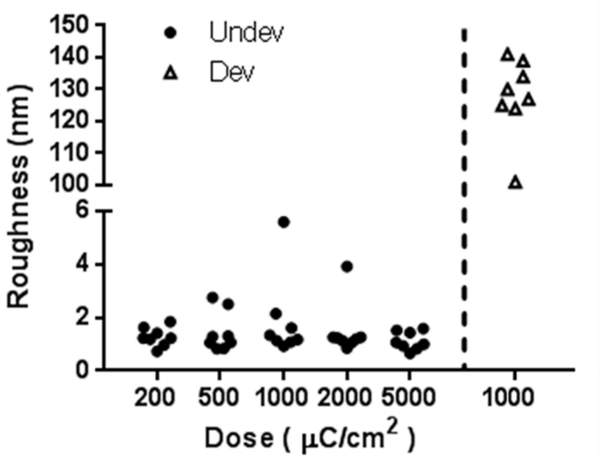

Figure S2: Surface roughness measurements of the undeveloped and the developed SU-8 film by AFAM.
